# Supplementary material for: From Coffee Waste to Active Ingredient for Cosmetic Applications
Source: Int J Mol Sci. 2023 May 10;24(10):8516. doi: 10.3390/ijms24108516 (PMC10218567; doi:10.3390/ijms24108516)
Supplement: Supplementary file 1 [file ijms-24-08516-s001.zip › ijms-2381917-supplementary.pdf]

Supplementary Table S1. Summary of high resolution LC/MS data.

CPC fractions	LC retention time (min)	Observed *m/z*	Elemental composition	Δppm	Annotation
F09	5.78	181.0726 [M+H]$^+$	$C_7H_9N_4O_2$	0.0	Theophylline or isomer
F09	6.23	181.0726 [M+H]$^+$	$C_7H_9N_4O_2$	0.0	Theophylline or isomer
F07 / F08 / F09 / F10 / F11	7.00	195.0900 [M+H]$^+$	$C_8H_{11}N_4O_2$	9.2	**Caffeine***
F11	7.93	193.0726 [M+H]$^+$	$C_8H_9N_4O_2$	0.0	Xanthine derivative
F09	8.24	211.1449	$C_{11}H_{19}N_2O_2$	0.9	*Not assigned*
F10 / F11	8.25	347.1860	$C_{20}H_{27}O_5$	0.6	*Not assigned*
F09 / F10 / F11	8.83	331.1910 [M+H]$^+$	$C_{20}H_{27}O_4$	0.3	**19-Norkaura-1,4(18)-diene-18-carboxaldehyde 16,17-dihydroxy-3-oxo* or isomer**
F09 / F10 / F11	10.09	331.1910 [M+H]$^+$	$C_{20}H_{27}O_4$	0.3	Bengalensol or isomer
F11	10.18	345.2065	$C_{21}H_{29}O_4$	-0.3	*Not assigned*
F11	10.40	329.1753	$C_{20}H_{25}O_4$	0.0	*Not assigned*
F09	10.57	329.1758	$C_{20}H_{25}O_4$	1.5	*Not assigned*
F10	10.84	313.1805 [M+H]$^+$	$C_{20}H_{25}O_3$	0.3	Dehydrokahweol or isomer
F10	11.10	331.1911	$C_{20}H_{27}O_4$	0.6	*Not assigned*
F09 / F11	11.27	313.1809 [M+H]$^+$	$C_{20}H_{25}O_3$	1.6	Dehydrokahweol or isomer
F03 / F05	11.65	569.4207 [M+H]$^+$	$C_{36}H_{57}O_5$	0.2	**19-Norkaura-1,4(18)-diene-18-carboxaldehyde 16,17-dihydroxy-3-oxo palmitate ester*** or isomer
F11	12.69	313.1808 [M+H]$^+$	$C_{20}H_{25}O_3$	1.3	Dehydrokahweol or isomer
F03 / F05	13.27	569.4206 [M+H]$^+$	$C_{36}H_{57}O_5$	0.0	**19-Norkaura-1,4(18)-diene-18-carboxaldehyde 16,17-dihydroxy-3-oxo palmitate ester*** or isomer
F02	13.54	597.4527 [M+H]$^+$	$C_{38}H_{61}O_5$	1.3	19-Norkaura-1,4(18)-diene-18-carboxaldehyde 16,17-dihydroxy-3-oxo stearate ester or isomer
F07	13.73	344.3166 [M+H]$^+$<br>687.6249 [2M+H]$^+$	$C_{20}H_{42}NO_3$<br>$C_{40}H_{83}N_2O_6$	0.3<br>-0.3	*Not assigned*
F10 / F11	15.13	566.4293 [M+H]$^+$	$C_{30}H_{56}N_5O_5$	2.1	*Not assigned*
F07 / F08	15.24	279.2326 [M+H]$^+$	$C_{18}H_{31}O_2$	0.7	Linolenic acid
F10 / F11	15.52	277.1805<br>181.1230	$C_{17}H_{25}O_3$	0.4	*Not assigned*
F02 / F05	15.54	429.3735 [M+H]$^+$	$C_{29}H_{49}O_2$	0.5	**Stigmastan-3,6-dione*** or isomer
F08 / F10	15.68	277.2173	$C_{18}H_{29}O_2$	1.8	*Not assigned*
F08 / F10	15.79	277.2171	$C_{18}H_{29}O_2$	1.1	*Not assigned*
F07	15.95	479.3740<br>365.2692	$C_{29}H_{51}O_5$	0.8	*Not assigned*
F03 / F04 / F06	16.06	429.3744	$C_{29}H_{49}O_2$	2.6	*Not assigned*
F08	16.19	279.2323<br>429.3732 [M+H]$^+$	$C_{18}H_{31}O_2$<br>$C_{29}H_{49}O_2$	-0.4<br>-0.2	**Stigmastan-3,6-dione*** or isomer

F04	16.32	585.4161	$C_{36}H_{57}O_6$	1.0	*Not assigned*
F03 / F04 / F05	16.53	599.4312	$C_{37}H_{59}O_6$	0.0	*Not assigned*
F02	16.56	419.3526	$C_{27}H_{47}O_3$	0.2	*Not assigned*
F04	17.11	585.4148	$C_{36}H_{57}O_6$	-1.2	*Not assigned*
F03 / F04 / F05	17.26	569.4208 $[M+H]^+$<br>279.0934 fragment	$C_{36}H_{57}O_5$	0.4	**19-Norkaura-1,4(18)-diene-18-carboxaldehyde 16,17-dihydroxy-3-oxo palmitate ester*** or isomer
F01	17.58	431.3527	$C_{28}H_{47}O_3$	0.5	*Not assigned*
F05	18.01	399.2903	$C_{26}H_{39}O_3$	1.0	*Not assigned*
F04	18.11	583.4359<br>279.0942	$C_{37}H_{59}O_5$	-0.5	*Not assigned*
F10 / F11	18.11	682.4642<br>887.5703	$C_{36}H_{64}N_3O_9$	-0.1	*Not assigned*
F03	18.42	613.4472	$C_{38}H_{61}O_6$	0.7	*Not assigned*
F05 / F06	19.64	609.4153 $[M+H]^+$<br>591.4044 $[M+H-H_2O]^+$<br>573.3932	$C_{38}H_{57}O_6$<br>$C_{38}H_{55}O_5$<br>$C_{38}H_{53}O_4$	-0.3<br>-0.8<br>-2.1	Diterpene fatty acid ester
F08	19.81	309.2795 $[M+H]^+$<br>279.0940	$C_{20}H_{37}O_2$<br><br>$C_{54}H_{79}O_9$	0.3<br><br>0.3	Linoleic acid ethyl ester
F06	20.20	607.4371 $[M+H]^+$	$C_{39}H_{59}O_5$	1.5	Methyl-diterpene linolenate
F02	20.50	611.4675 $[M+H]^+$	$C_{39}H_{63}O_5$	-0.2	Methyl-diterpene linoleate
F03 / F04 / F10	20.53	583.4368 $[M+H]^+$	$C_{37}H_{59}O_5$	1.0	Methyl-diterpene palmitate
F07	20.78	308.2957	$C_{18}H_{38}NO$	1.3	*Not assigned*
F06	21.10	623.4316<br>279.0936	$C_{39}H_{59}O_6$	0.6	*Not assigned*
F04 / F05	21.32	609.4887	$C_{40}H_{65}O_4$	0.7	**Cafestol gadoleinate ester***
F08	21.53	407.3164	$C_{25}H_{43}O_4$	0.7	*Not assigned*
F05	21.66	593.4210 $[M+H]^+$<br>575.4108 $[M+H-H_2O]^+$	$C_{38}H_{57}O_5$		**19-Norkaura-1,4(18)-diene-18-carboxaldehyde 16,17-dihydroxy-3-oxo linoleate ester *** or isomer
F06	21.88	310.3115	$C_{20}H_{40}NO$	1.6	*Not assigned*
F05	21.89	585.4162	$C_{36}H_{57}O_6$	1.2	*Not assigned*
F05	22.42	625.4831 $[M+H]^+$<br>427.3580	$C_{40}H_{65}O_5$<br>$C_{29}H_{47}O_2$	-0.2<br>0.9	**19-Norkaura-1,4(18)-diene-18-carboxaldehyde 16,17-dihydroxy-3-oxo gadoleinate ester ***
F02	22.43	393.3157	$C_{28}H_{41}O$	0.0	Sterol

*also identified by NMR
